# Exploring polygenic contributors to subgroups of comorbid conditions in autism spectrum disorder

**DOI:** 10.1038/s41598-022-07399-7

**Published:** 2022-03-01

**Authors:** Louis Klein, Shannon D’Urso, Valsamma Eapen, Liang-Dar Hwang, Ping-I Lin

**Affiliations:** 1 School of Psychiatry, University of New South Wales, Sydney, NSW Australia; 2 Mental Health Research Unit, South Western Sydney Local Health District, Liverpool, Australia; 3 Institute for Molecular Bioscience, The University of Queensland, Brisbane, QLD Australia

**Keywords:** Genetics, Medical research, Risk factors, Signs and symptoms

## Abstract

Individuals with autism spectrum disorder (ASD) have heterogeneous comorbid conditions. This study examined whether comorbid conditions in ASD are associated with polygenic risk scores (PRS) of ASD or PRS of comorbid conditions in non-ASD specific populations. Genome-wide single nucleotide polymorphism (SNP) data were obtained from 1386 patients with ASD from the Autism Genetic Resource Exchange (AGRE) study. After excluding individuals with missing clinical information concerning comorbid conditions, a total of 707 patients were included in the study. A total of 18 subgroups of comorbid conditions (‘topics’) were identified using a machine learning algorithm, topic modeling. PRS for ASD were computed using a genome-wide association meta-analysis of 18,381 cases and 27,969 controls. From these 18 topics, Topic 6 (over-represented by allergies) (*p* = 1.72 × 10^−3^) and Topic 17 (over-represented by sensory processing issues such as low pain tolerance) (*p* = 0.037) were associated with PRS of ASD. The associations between these two topics and the multi-locus contributors to their corresponding comorbid conditions based on non-ASD specific populations were further explored. The results suggest that these two topics were not associated with the PRS of allergies and chronic pain disorder, respectively. Note that characteristics of the present AGRE sample and those samples used in the original GWAS for ASD, allergies, and chronic pain disorder, may differ due to significant clinical heterogeneity that exists in the ASD population. Additionally, the AGRE sample may be underpowered and therefore insensitive to weak PRS associations due to a relatively small sample size. Findings imply that susceptibility genes of ASD may contribute more to the occurrence of allergies and sensory processing issues in individuals with ASD, compared with the susceptibility genes for their corresponding phenotypes in non-ASD individuals. Since these comorbid conditions (i.e., allergies and pain sensory issues) may not be attributable to the corresponding comorbidity-specific biological factors in non-ASD individuals, clinical management for these comorbid conditions may still depend on treatments for core symptoms of ASD.

## Introduction

Psychiatric and medical comorbidities are a norm rather than an exception in autism spectrum disorder (ASD); a complex neurodevelopmental disorder characterized by social communication deficits and restricted/repetitive behaviors^1^. The importance of understanding medical comorbidities of ASD cannot be understated^2^. Appropriate management of comorbid medical conditions may lead to quality of life improvement for both children and their parents^3^. In this regard, understanding of shared etiologies—including potential genetic factors^4,5^—for ASD and comorbid conditions may be critical in management decisions. While research into the medical comorbidities of ASD has been ongoing^2^, research into the genetic bases of ASD’s psychiatric comorbidities is only now getting underway^6–8^. Understanding how genetic factors contribute to the comorbidities may provide novel insight into molecular mechanisms underlying heterogeneous clinical features of individuals with ASD. Such genetic components could be used to subgroup patients with ASD to generate clinical subtypes that reflect biological differences, which might provide opportunities for individualized treatment options^9^.

Prior studies that have investigated genetic contributions for comorbidities in ASD have implemented several different approaches. For instance, David et al. conducted an automated extraction of genes associated with ASD and its comorbid disorders, finding 1031 genes associated with ASD—262 of these genes were involved in ASD only—while the remaining 779 genes were also associated with other comorbid disorders^10^. Their study results suggest that the majority of candidate genes for ASD have pleiotropic effects. Diaz-Beltran et al. used a two-fold systems biology approach to perform a comparative analysis of ASD with 31 frequently encountered comorbid disorders and determined a multi-comorbidity subtype of ASD, which led to the discovery of novel candidate genes of ASD^11^. Tylee et al. used data from previous genome-wide association studies (GWAS) to determine whether commonly varying single nucleotide polymorphisms (SNPs) are shared between psychiatric and immune-related phenotypes, and found that ASD is most likely to correlate with allergy rather than all other major psychiatric disorders^12^. There is also evidence to suggest that ASD is often comorbid with the perception of pain, with ASD patients having a higher threshold of pain^13,14^. In this regard, Johnson et al. conducted a linkage disequilibrium score regression using GWAS associations for ASD and chronic pain to show that the two traits are genetically correlated^15^. Finally, a recent study used polygenic risk scores (PRS) derived from five psychiatric disorders, such as schizophrenia, major depressive disorder, attention deficit hyperactivity disorder, obsessive–compulsive disorders, and anxiety, and found that the polygenic contributions could distinguish Asperger syndrome (a diagnostic category in the Diagnostic and Statistical Manual (DSM) 4th Edition although this term is no longer used in the updated DSM-5) from individuals with other non-Asperger subtypes of ASD^16,17^.

Despite these advances in understanding the relationships between the genetic bases for ASD and associated comorbidities, it is clear that these approaches require further development to fully understand the role of comorbid genetic risk within ASD. In the present study, we used topic modeling—a spatial clustering process tolerant of feature sparsity (for e.g., diagnostic features in low prevalence medical conditions)—to identify clusters of comorbid conditions. Similar methods were utilized in an earlier study by McCoy and colleagues to investigate which types of comorbid conditions are attributable to polygenic loading of major depressive disorder^18^. The authors observed that using standard phenome-wide association studies (PheWAS) to test across all possible predictors such as individual risk variants or genome-wide variants increases Type I error as the process involves testing against all diagnostic codes (e.g. 1500+ codes), or Type II error as the approach needs to correct for all these codes. This approach also does not take into consideration the correlation between individual diagnosis codes and the inconsistency and lack of reliability of the codes per se. By first using topic modeling on a corpus of ICD-9 diagnostic codes, McCoy et al. were able to reduce the dimensionality of possible MDD comorbidity and thereby also mitigate risk of imprecision when examining associations with genetic indicants. However, it is important to note several limitations to this approach including that it cannot distinguish whether depression and associated co-morbidities were caused by shared versus unique but convergent genetic factors. Given that the number of topics was chosen arbitrarily, it is likely that an alternative number of topics would yield different results in terms of cluster composition. Moreover, it is possible to optimize the selection of the number of topics to cluster upon using a range of semantic and statistical indices developed by researchers in the applied topic modeling literature.

The present study extends the topic modeling approach applied by McCoy and colleagues by (1) implementing a novel method for constructing document data from epidemiological data; (2) determining the number of topics was determined by a semi-unsupervised process that maximizes the trade-off between topic sensitivity and term specificity, and (3) jointly investigating associations between topics of ASD and three sets of PRSs of ASD and its clinically co-morbid conditions of interest, namely, chronic pain and allergies, respectively.

## Methods

All methods were carried out in accordance with relevant guidelines and regulations.

### Sample description

The discovery sample of ASD comprised the whole-genome genotypic data retrieved from the Autism Genetic Resource Exchange (AGRE), of which the subject recruitment has been described elsewhere^19^. Briefly, AGRE is a joint effort of the Cure Autism Now (CAN) Foundation and the Human Biological Data Interchange (HBDI). The diagnosis was made by all of the NIH autism collaborative groups using the Autism Diagnostic Interview-Revised (ADI-R)^20^ and the Autism Diagnostic Observational Schedule (ADOS)^21^. We have downloaded the clinical and SNP data (generated by the Affymetrix SNP 5.0 platform). We implemented the same data-cleaning algorithm used in the discovery sample. A total of 325,971 valid SNPs for 1387 subjects, 97.3% had European ancestry^22^, diagnosed with ASD were obtained. The final dataset consisting of 707 subjects with comorbid physical and psychiatric features as well as perinatal factors were used to examine the patterns of comorbid conditions in the present study.

### Data preparation

Data preparation was performed using the R software v3.6.1^23^ within the RStudio integrated development environment^24^. Full reproducible code and session information is available upon request.

#### Pseudo-EMRs

Documents were constructed as electronic medical record (EMR) analogues on the basis of a multistep process. Each psychiatric feature (for e.g., symptoms, diagnoses, historical presentations) was considered a candidate term for inclusion in the ‘pseudo-EMR’ for each subject. Categorical data were collapsed into the presence versus absence of features. Continuous data were then dichotomized using *k*-means clustering (i.e. *k* = 2)^25,26^. Some continuous features required special consideration in relation to dichotomization, for e.g., features relating to developmental delays for which delay thresholds were drawn from clinical literature. Features were excluded where counts per feature were less than or equal to 1, as these were considered prohibitively sparse. Following dichotomization, psychiatric features were transcoded into labels indicating the presence of features by subject. Hyphenation was used to coerce features with complex clinical descriptions into single “terms” (e.g., ‘floppy infant’ became ‘infant-floppy’). This is done to ensure that subsequent topic modeling did not tokenize features in ways disrupting correspondence with the observed data.

#### Topic modelling

**Figure 1:**
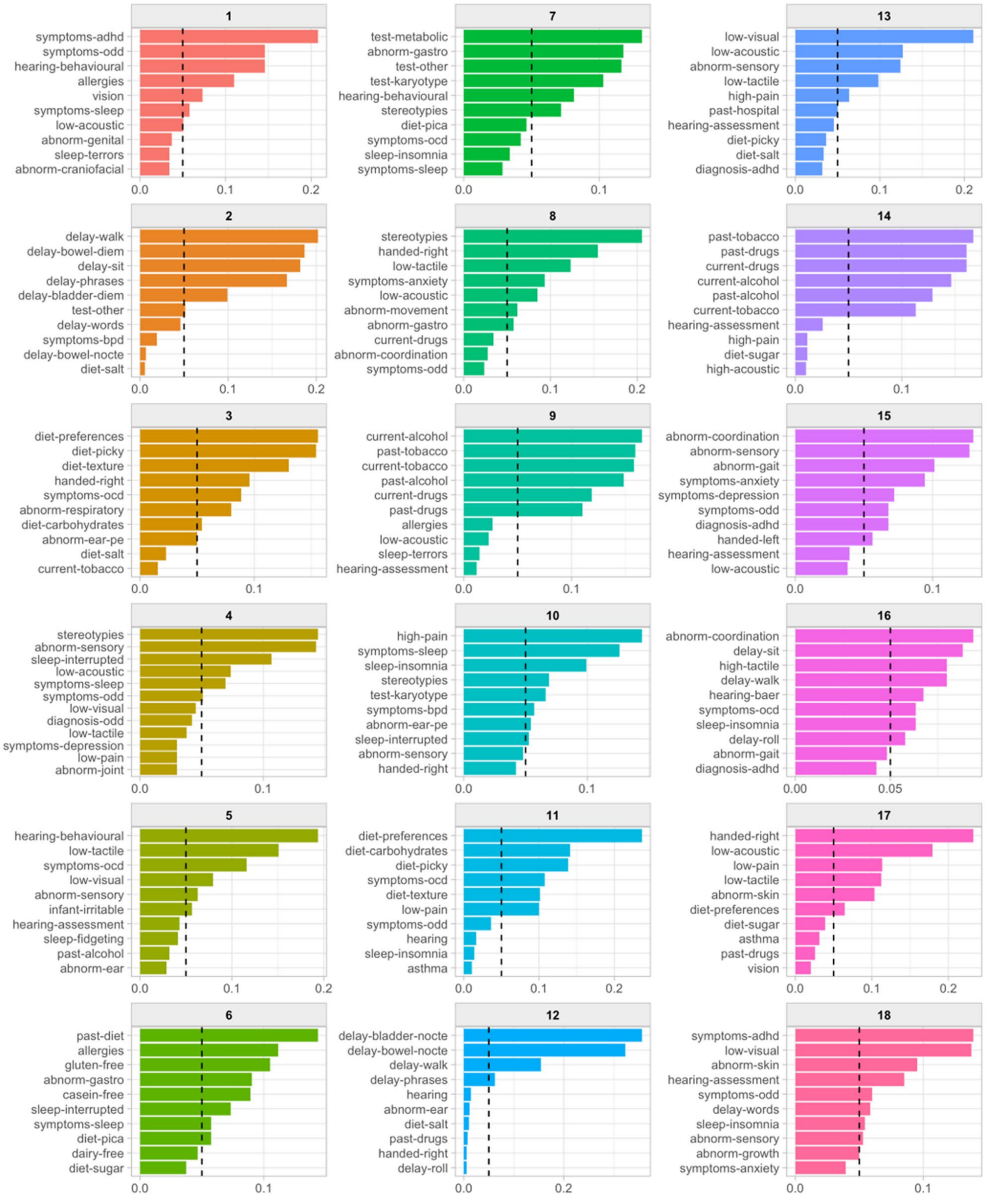
‘Terms-to-topic’ probabilities for patients with autism spectrum disorder (ASD). The figure illustrates the topics (i.e., clusters of comorbid conditions) and their over-represented terms (i.e., comorbid conditions). Names for each panel indicate the topic number from which associated data are drawn. *x*-axes indicate beta weights (*β*), signifying the probability of each term falling under the specific topic. *y*-axes indicate the most probable (*n* = 10) terms rank-ordered by *β* weight. Dashed lines indicate *β* = 0.05.

The optimal number of topics to model was explored prior to topic modeling^27^. Briefly, topic modeling, such as Latent Dirichlet Allocation (LDA), is used to identify ‘topics’ (i.e., clusters of comorbid conditions in the current study) that occur in a collection of documents^28^. Accordingly, a parallel process was run over a range of candidate models in order to evaluate the relationship between number of topics and model diagnostics according to recommendation by Chang et al. (Supplementary Fig. 1)^29^. The outcome of this procedure was the selection of 18 as the optimal number of topics to model given the observed data. LDA was then applied using Gibbs sampling with the following settings: 2000 iterations were performed with 500 iterations for thinning and a burn-in of 1000 iterations. Symmetric Dirichlet priors were applied to ensure topics would be well-separated; *α* = 0.1, *β* = 0.01^30^. A threshold of 16 terms (i.e., comorbid conditions) per topic was chosen following inspection of the resulting topic models (for term-to-topic probabilities, see Fig. 1. Topic modeling was conducted using the ‘topicmodels’ package v0.12^31^.

### Genetic association analyses

#### Genotype and imputation

All subjects were genotyped using the Affymetrix GeneChip Human Mapping 500 K Array. We retained subjects with genotyping call rates exceeding 90% and single nucleotide polymorphisms (SNPs) with a call rate of 90% or greater, and Hardy–Weinberg equilibrium *p*-value > 1 × 10^−6^. We remapped the raw genotype data from the GRCh35 to GRCh37 and conducted quality control by removing SNPs (1) with ambiguous alleles, (2) with > 0.2 allele frequency difference from the reference panel, and (3) not available in the reference panel using the Pre-imputations checks toolbox (https://www.well.ox.ac.uk/~wrayner/tools/) and the 1000 Genome European reference panel. Genotypes were next imputed using the Michigan Imputation Server implementing Minimac4^32^, based on the European subset from the 1000 Genomes Phase 3 v5 (GRCh37/hg19) as reference panel with an imputation filter of *R*^2^ > 0.3. Phasing of haplotypes was conducted using ‘Eagle’ v2.4^33^.

#### Polygenic risk (PRS) calculation

We generated polygenic risk scores (PRS) for ASD (*n* = 18,381 cases and 27,969 controls)^34^, allergic disease (*n* = 180,128 cases and 180,709 controls)^6^ and chronic pain (*n* = 387,649)^15^, using seven tranches of SNPs (1 × 10^−2^, 1 × 10^−3^, 1 × 10^−4^, 1 × 10^−5^, 1 × 10^−6^, 1 × 10^−7^, 5 × 10^−8^, labelled as S2–S8) drawn from recent GWAS. The value for each *p*-value tranche represents the maximum *p*-value that is included in that tranche. This list was linkage-disequilibrium pruned using the ‘clump()’ function as implemented in ‘PLINK’ v1.9, with a 250 kb window and minimum *R*^2^ set at 0.5 by default^35^.

### Statistical analyses

The relationship between PRS tranches and topics across ASD, allergic disease and chronic pain were tested using a linear regression model. All model topics were inverse normal transformed prior to analysis because they were not normally distributed. Covariates included age, sex, and the first five genetic principal components. We conducted analysis of variance (ANOVA) tests to compare the fits of models. Linear regression and ANOVA tests were conducted using the R language v3.6.1^23^ within the RStudio IDE^32^. Further, we estimated the proportion of the variance of the dependent variable (i.e., topics of comorbid conditions) that could be explained by each predictor (i.e., PRS specific to ASD, PRS specific to the corresponding topic, age, and gender) using partial and semi-partial correlation coefficients of a specified predictor—which were used to compare relative contributions of PRS specific to different phenotypes to the topics. Finally, we examined genetic correlations between ASD and comorbid conditions associated with PRS specific to ASD based on the lists of candidate genes extracted using the web tool, Genepanel.iobio^36^. The correlation was inferred based on the probability of detecting significant phenotype–phenotype associations by random chance calculated using the hypergeometric distribution^37^.

## Results

**Figure 2:**
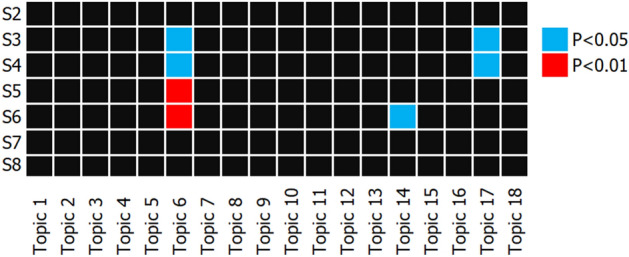
Heatmap of the associations between all topics and polygenic risk score (PRS) tranches of autism spectrum disorder (ASD). Topics were inverse-normal transformed prior to linear regression modelling with age, sex, and first five genetic principle components included as predictors. The figure shows that associations between the topics (i.e., clusters of comorbid conditions) and PRS tranches indicating polygenic loads for ASD. Topic 6 is over-represented by terms relating to allergies while Topic 17 is over-represented by sensory processing issues.

We found a strong association between *PRS*_*ASD*_ and the Topic 6, the main allergy factor (the first five terms: *past-diet*, *allergies*, *gluten-free*, *abnormal-gastro*, *casein-free*) (Fig. 2). The strongest associated *PRS*_*ASD*_ tranche S5 (*p* = 1.72 × 10^−3^) accounted for 1.41% of the variance in the Topic 6. We also found that Topic 17 (first 5 terms: *handed-right*, *low-acoustic*, *low-pain*, *low-tactile*, *abnorm-skin*, *diet-preferences*; minimum *p* = 0.037 for S4) accounted for 0.80% of the variance. Topic 14 was over-represented by maternal substance use and hence was excluded from the subsequent analyses (see Supplementary Table 1 for full results).

**Figure 3:**
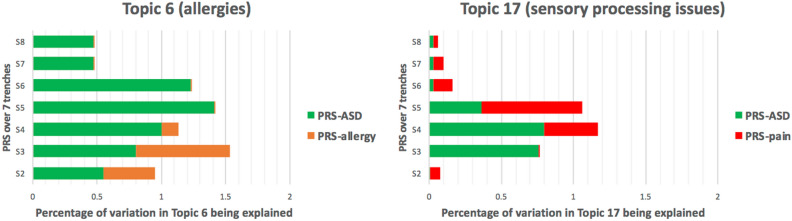
Relative contributions of polygenic risk scores (PRS) to the two topics of comorbid conditions. Relative contributions were calculated using the squared values from semi-parametric correlation tests. Topic 6 is over-represented by allergies while Topic 17 is over-represented by sensory processing issues. *PRS*_*ASD*_ indicates the PRS values derived from the GWAS of ASD, *PRS*_*allergy*_ indicates the PRS values derived from the GWAS of allergies, and *PRS*_*pain*_ indicates the PRS values derived from the GWAS of chronic pain disorder.

We further assessed the association between Topic 6 and *PRS*_*allergy*_ and found no evidence for an association (minimum *p* = 0.083 for *PRS*_*allergy*_ S2). When fitting both *PRS*_*ASD*_ and *PRS*_*allergy*_ into the model, the strengths of the association for the two PRSs slightly increased (*PRS*_*ASD*_ S5: *p* = 1.48 × 10^−3^; *PRS*_*allergy*_ S2: *p* = 0.070), but the overall model fit did not improve compared against the *PRS*_*ASD*_ only model (*p*_*ANOVA*_ = 0.070). Similarly, no evidence was found for an association between the Topic 17 and *PRS*_*pain*_ (minimum *p* = 0.052 for S5). However, combining *PRS*_*ASD*_ and *PRS*_*pain*_ improved the prediction of Topic 17 (*p*_*ANOVA*_ = 0.031; the two PRSs together accounts for 1.8% of the variance) and boosted the strength of associations for each of the two PRSs (*PRS*_*ASD*_ S4: *p* = 0.022;  *PRS*_*pain*_ S5: *p* = 0.031). Overall, *PRS*_*ASD*_ contributed to a greater degree to the variance in topic 6 than *PRS*_*allergy*_, and *PRS*_*ASD*_ contributed to a greater degree to the variance in Topic 17 than *PRS*_*pain*_ (see Fig. 3).

**Figure 4:**
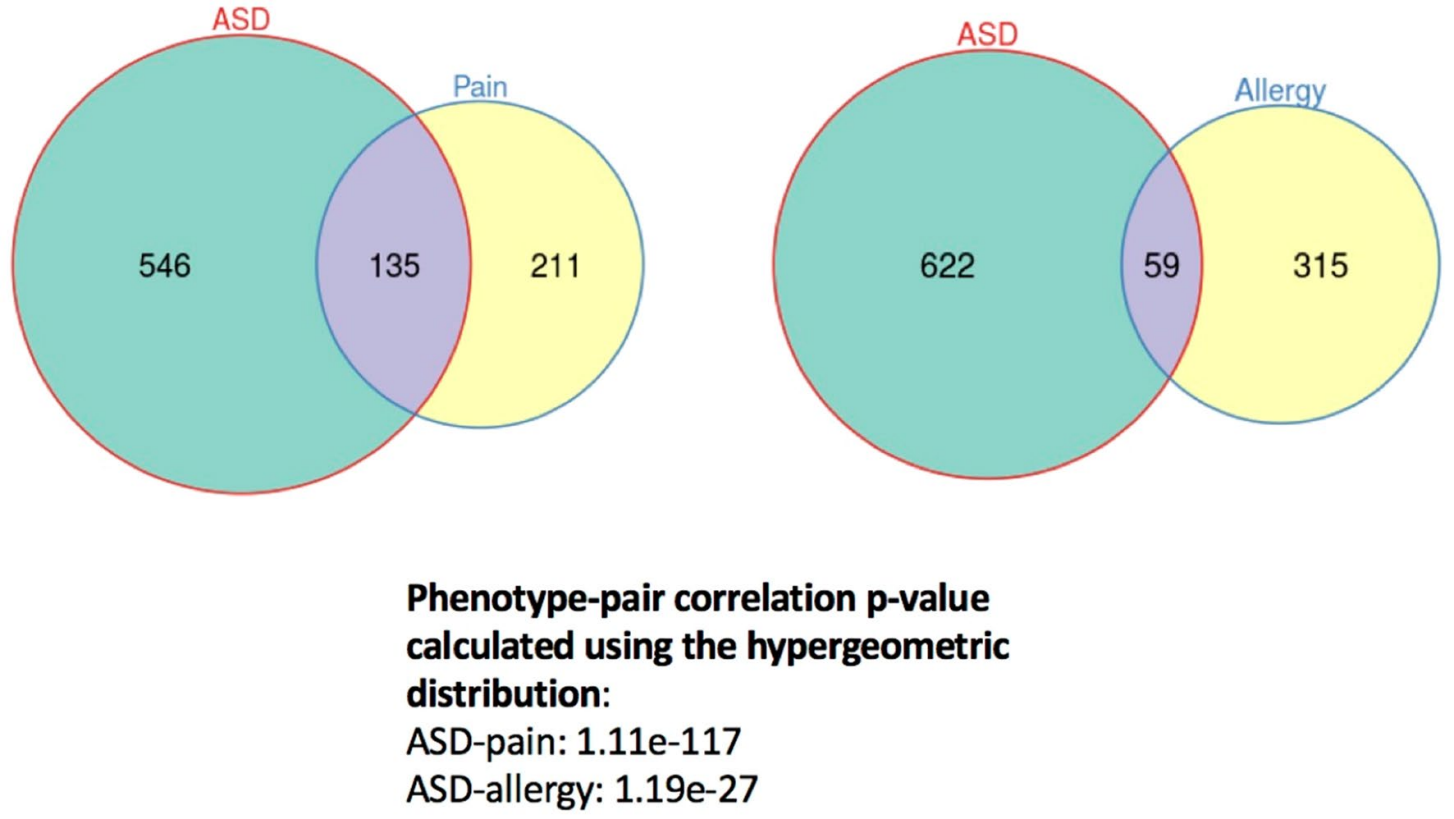
Genetic correlations between autism spectrum disorder (ASD) and the other two comorbid phenotypes (allergy, pain). The numbers within each circle indicate the number of candidate genes corresponding to different phenotypes (ASD, pain sensory conditions, and allergy-related conditions) based on the literature, and the size of the circles are proportional to the number of candidate genes in each set of Venn diagram.

We have identified 681 candidate genes for ASD, 374 candidate genes for allergies, and 346 candidate genes for pain-related disorder (e.g., abnormality in pain sensory perception or impaired pain sensory perception) (see Fig. 4). There were 59 overlapping genes between the lists of candidate genes for ASD and allergies, and 135 overlapping genes between the lists of candidate genes for ASD and pain-related disorder. The *p*-value for the correlation between ASD and allergies was 1.19 × 10^−2^^7^, while the *p*-value for the correlation between ASD and pain-related disorder was 1.11 × 10^−1^^17^.

## Discussion

The current study shows that polygenic loading in ASD may play a larger role in certain subgroups of comorbid conditions in ASD, such as allergy-related conditions, and sensory processing issues (e.g., low pain tolerance) than other subgroups of comorbid conditions. We further found that these two subgroups (i.e., topics) of comorbid conditions could not be attributed to PRS of either allergies or chronic pain disorder. These findings suggest that ASD-associated genetic variants could contribute to ASD-related comorbidities including allergies and sensory processing issues such as low pain tolerance. In other words, these two types of comorbidities may share a proportion of risk variants with ASD. Notably, we found that both of these two subgroups (i.e., topics) of comorbid conditions in individuals with ASD were not correlated with their corresponding PRS constructed using GWAS of non-ASD specific individuals.

Nevertheless, although both pain and pain sensory issues may share susceptibility genes with ASD^38,39^, our findings suggest that ASD may be more likely to correlate with abnormality in pain tolerance at the gene level compared to allergies. This is consistent with our findings that *PRS*_*pain*_ might contribute, at least to a slightly higher degree, to the corresponding topic enriched with sensory processing issues than that of allergies and that combining *PRS*_*ASD*_ and *PRS*_*pain*_ strengthened the association with the corresponding comorbid conditions. These findings, to our knowledge, have not yet been published in other studies.

The current study has several limitations. First, characteristics of the present AGRE sample and those samples used in the original GWAS for ASD, allergies, and chronic pain disorder, may differ. For example, there may be significant clinical heterogeneity in the ASD population. This is further compounded by the significant changes in the diagnostic classification that has occurred in the past decade that would have potentially changed the ascertainment and phenotypic characterization of ASD. For example, in 2013 the Diagnostic and Statistical Manual 5th edition combined the different subcategories such as Asperger syndrome and disintegrative disorder etc. and collapsed it into a single category of ASD and further changes were made to the diagnostic criteria such as the inclusion of sensory issues^16^, all of which would have impacted the ascertainment of the sample and the phenotypic characterization. Second, the clinical data that relied on self-report information, might not provide the most accurate or robust information about comorbid conditions due to false clinical assumptions. This may hence lead to biased results that make it difficult to replicate the findings in independent populations. This could partially contribute to the failure to observe allergy-related and pain-related polygenic loads associated with the allergy-related topic and pain-related topic, respectively, in our sample. Further, based on the developmental stage in which the sample was recruited there may be differences in the phenotypic characterization as development is a dynamic process and phenotypic manifestations may emerge later in life or change over the life course. Therefore, the reference GWAS studies of ASD, allergies, and chronic pain disorder, which were based on samples, might not provide unbiased references for the test sample of ASD based on pediatric samples. Specifically, some comorbid conditions may only manifest after certain ages and hence reference effect sizes based on the adult sample may lead to biased prediction using PRS values in the test sample consisting of children. The null association between *PRS*_*pain*_ and Topic 17 may indicate that age also played a substantial role in Topic 17; a cluster of comorbid conditions over-represented by sensory processing issues such as low pain tolerance, despite adjusting for age in the model. Second, each of the topics of comorbidities refers to a cluster of various correlated but distinct phenotypes, where the genetic architectures may not be well captured by PRS derived from a GWAS study of one single phenotype. Third, predictive values of PRS may substantially decrease if training (i.e. reference cohort) and testing data (i.e. scored cohort) sets are drawn from different populations^40^. Fourth, the AGRE sample may be underpowered to detect weak PRS associations due to its small sample size.

Nonetheless, our research has several noteworthy strengths. First and foremost, we successfully applied the methodology employed by McCoy and colleagues to comorbid conditions associated with ASD, which is an arguably more complex psychiatric category for diagnosis and detection than MDD^41,42^; the domain of mental illness in which this approach was first tested. Second, our implementation used a semi-supervised approach to the selection for the optimal number of topics based upon recent advances in the applied topic modeling literature. Thirdly, the use of a phenotypically well characterized sample to identify ASD subgroups using topic modeling is a substantial strength of the study. Finally, the way in which pseudo-EMRs were generated on the basis of clinical/diagnostic data for the purpose of topic modeling is novel and may well be useful in other analytic contexts.

In summary, the current findings suggest that the occurrence of two subgroups of ASD-related comorbidities—allergies and chronic pain—may be driven by shared underlying genetic risk factors for ASD. Notably, these two types of comorbid conditions could not be attributable to genetic variants associated with either allergies or chronic pain disorder in non-ASD populations. Such findings suggest that genetic mechanisms of certain comorbid conditions such as allergies and sensory processing issues in patients with ASD could differ from those of the non-ASD population. Further, it is possible that there is a subgroup where the specific co-morbidities may indicate an alternate converging process such as a common immune pathway^43^. In this regard, immune system deficiencies and immune dysregulation in ASD may result in a wide variety of co-morbidities such as allergic sensitivities, asthma, rashes, gastro intestinal and skin sensitivities as well as sensory issues. Thus, the findings of genetic contributors for comorbid conditions in ASD may inform clinical management strategies. For example, treatment of comorbid allergies in persons diagnosed with ASD may still depend on the clinical management of core symptoms of ASD rather than allergy-specific therapies despite previously reported genetic correlations between ASD and allergies^12^. On the other hand, the pharmacological treatment for ASD may need to take underlying immune related issues into account. Further, although emerging evidence suggests that peripheral somatosensory neurons are involved in tactile-related phenotypes in ASD^44,45^, genetic variants associated with pain disorder seems at best to only play a limited role in abnormalities in pain sensory perception, in children with ASD. This deserves further exploration through research involving larger population samples as better understanding of the underlying pathogenetic mechanisms involved in comorbid conditions in ASD may have clinical implications in the comprehensive assessment and management of these patients.

## Supplementary Information

Supplementary Information.
