# Supplementary material for: Exploring polygenic contributors to subgroups of comorbid conditions in autism spectrum disorder
Source: Sci Rep. 2022 Mar 1;12:3416. doi: 10.1038/s41598-022-07399-7 (PMC8888546; doi:10.1038/s41598-022-07399-7)
Supplement: Supplementary file 1 — Supplementary Information. [file 41598_2022_7399_MOESM1_ESM.docx]

**Supplementary Files**

**Supplementary Figure 1 — *Evaluation of various model diagnostics in relation to selecting the optimal number of topics to model given the observed data***Top left: Held-out likelihood indicates perplexity (higher is better). Top right: Lower bound indicates model convergence (higher is better). Bottom left: Residuals indicate model saturation (lower is better). Bottom right: Semantic coherence indicates co-occurrence of probable terms per topic (higher is better).


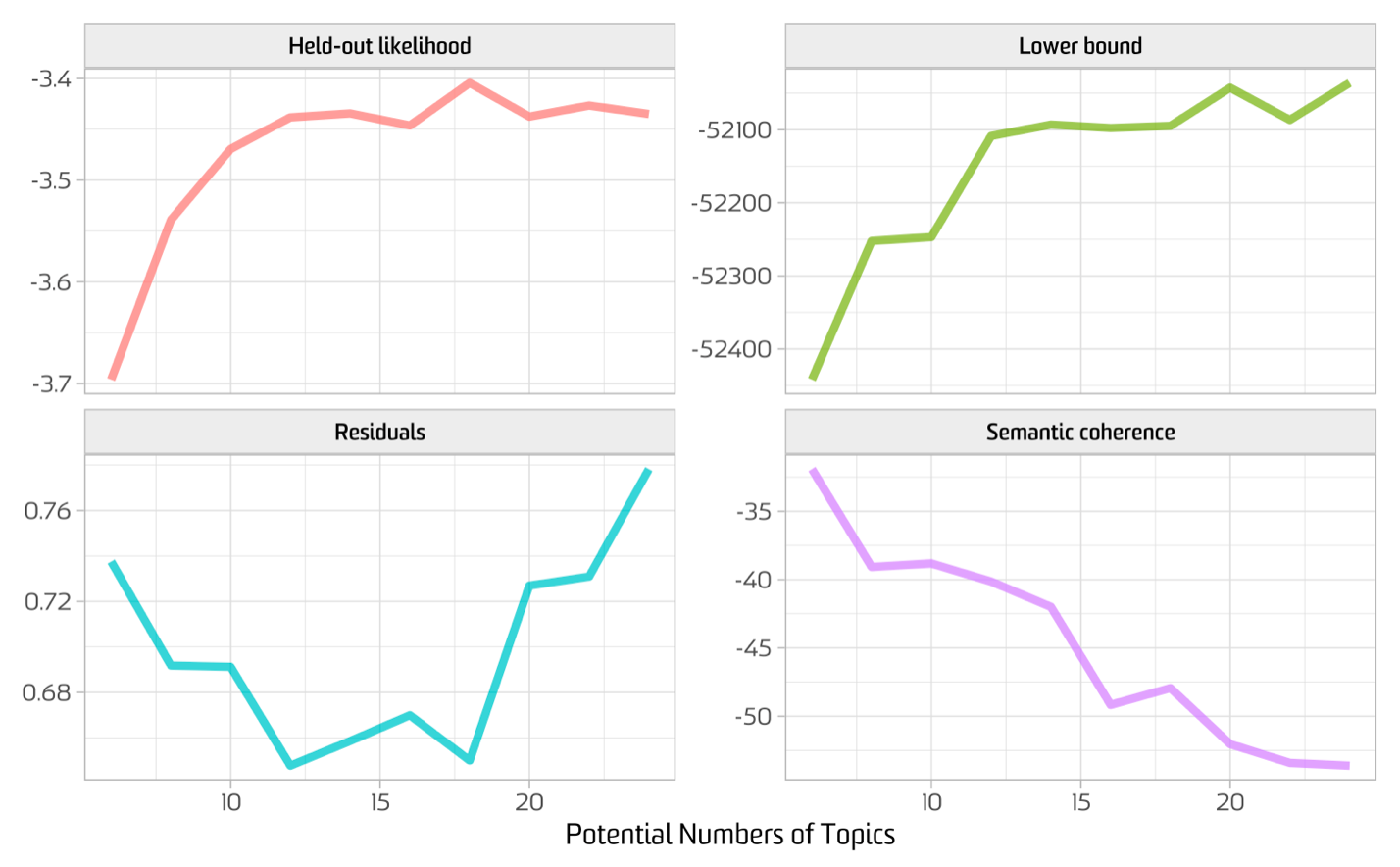


**Supplementary Table** **1 — *Association between all topics and polygenic risk score (PRS) tranches of autism spectrum disorder (ASD)***Seven tranches of SNPs (1x10−2, 1x10−3, 1x10−4, 1x10−5, 1x10−6, 1x10−7, 5x10−8) are labelled as S2-S8.
